# Verletzung des vorderen Kreuzbandes beim Erwachsenen: Diagnostik und Therapie

**DOI:** 10.1007/s00132-020-03997-3

**Published:** 2020-10-21

**Authors:** L. Kohn, E. Rembeck, A. Rauch

**Affiliations:** 1 Klinik für Unfallchirurgie und Orthopädie, Krankenhaus Landshut-Achdorf, Landshut, Deutschland; 2 Sporttraumatologie und Kniechirurgie, ATOS-Klinik München, ECOM – Praxis für Orthopädie, Sportmedizin & Unfallchirurgie, München, Deutschland

**Keywords:** Knieverletzung, Knieinstabilität, Vordere Kreuzbandruptur, Vordere Kreuzbandersatzplastik, Vordere Kreuzbandrefixation, Knee injury, Knee instability, Anterior cruciate ligament rupture, Anterior cruciate ligament replacement, Anterior cruciate ligament refixation

## Abstract

Das vordere Kreuzband (VKB) ist gemeinsam mit dem hinteren Kreuzband ein zentraler Stabilisator des Kniegelenkes. Es stabilisiert die Tibia gegen eine vermehrte Translation nach ventral sowie gegen eine vermehrte Innenrotation. Mit einer Inzidenz von 46/100.000 zählen Risse des vorderen Kreuzbandes zu den häufigsten Sportverletzungen in Deutschland. Neue Präventionsprogramme können das Risiko einer Kreuzbandverletzung verringern. Bei jungen und sportlich aktiven Patienten wird die operative Behandlung empfohlen, wodurch auch das Risiko von relevanten Meniskus- und Knorpelverletzungen in der Folge verringert werden kann. Standard der operativen Therapie in Deutschland ist die vordere Kreuzbandersatzbandplastik mittels autologer Sehne. In ausgewählten Fällen zeigt der operative Erhalt des vorderen Kreuzbandes durch arthroskopische Refixation gute Ergebnisse. Anstelle der bisherigen rein zeitbasierten Rehabilitation werden zunehmend funktionsbasierte Kriterien in die Nachbehandlung mit einbezogen.

## Abstract (en)

The anterior cruciate ligament (ACL) together with the posterior cruciate ligament is the central stabilizer of the knee. It stabilizes the tibia against increased anterior translation and internal rotation. With an incidence of 46/100,000 ACL tears are among the most common sports injuries in Germany. New prevention programs can reduce the risk of ACL injuries. Surgical treatment is recommended for young and athletic patients as it can also reduce the risk of further relevant injuries of the meniscus and cartilage. The standard of surgical treatment in Germany is the ACL reconstruction with an autologous tendon graft. In selected cases, the preservation of the ACL by arthroscopic refixation shows good results. Instead of the previously used purely time-based rehabilitation, function-based criteria are increasingly being included in the aftercare.

## Lernziele

Nach der Lektüre dieses Beitrags …

- kennen Sie die anatomischen Besonderheiten des vorderen Kreuzbandes,
- sind Ihnen die Methoden zur Diagnostik auch im Hinblick auf Begleitpathologien bekannt,
- wissen Sie, wann welche Therapie durchgeführt werden sollte,
- haben Sie einen Überblick über die gängigsten operativen Therapieverfahren.

## Einleitung

Jährlich über 40.000 durchgeführte VKB(vordere Kreuzband)-Operationen in Deutschland zeigen die Relevanz der VKB-Rupturen in der heutigen Orthopädie. Dem wird auch durch die ständige Weiterentwicklung des anatomischen und biomechanischen Wissens, der Operationstechniken und ganz aktuell auch der Prävention und Nachbehandlung Rechnung getragen. Somit untersteht die vordere Kreuzbandchirurgie einem ständigen Wandel mit dem Ziel, die Patienten wieder sicher und dauerhaft auf ihr altes sportliches Niveau zu bringen und eine Arthrose zu vermeiden.

Dieser Übersichtsbeitrag gibt einen Einblick in die neuesten anatomischen sowie biomechanischen Erkenntnisse, die daraus resultierenden Überlegungen zur weiteren Therapie sowie zu den neuesten Empfehlungen in der Nachbehandlung und Prävention.

## Anatomie

Die Kenntnis über die exakte Anatomie des VKB ist Grundvoraussetzung für eine **anatomische Rekonstruktion**

Anatomische Rekonstruktion.

Im Laufe der Jahre kam es immer wieder zu neuen Erkenntnissen und somit zu einer Anpassung der Operationstechniken. Das VKB verläuft schräg durch die Fossa intercondylaris von der Innenseite des lateralen Femurkondylus bis zur Eminentia intercondylaris. Aktuelle Arbeiten konnten zeigen, dass der femorale Ursprung des VKB halbmondförmig ist. Begrenzt wird er ventral von der sog. lateralen interkondylaren Kante („resident’s ridge“) und nach posterior von dem konvexen Knochen-Knorpel-Übergang der posterioren lateralen Femurkondyle. Von dem bandförmigen Hauptansatz, der unmittelbar posterior der „resident’s ridge“ in Verlängerung des posterioren Femurkortex liegt, strahlen zusätzliche Fasern nach dorsal fächerförmig aus [1, 2, 3].

Das VKB ist eine komplexe Struktur aus **dichtem Bindegewebe**

Dichtes Bindegewebe

mit einer Vielzahl von Fasern. In seinem Verlauf zeigt sich das VKB als flache **bandförmige**

Bandförmig

Struktur und nicht oval geformt. Es besteht in der Literatur weitestgehend Akzeptanz, dass es funktionell in ein anteromediales und ein posterolaterales Bündel unterteilt werden kann. Die Nomenklatur anteromedial und posterolateral richtet sich hier nach der Lage der tibialen Insertion [4, 5, 6, 7]. Das **anteromediale Bündel**

Anteromediale Bündel

inseriert dabei unmittelbar medial des medialen Interkondylarhöckers und das **posterolaterale Bündel**

Posterolaterale Bündel

nahe des lateralen Interkondylarhöckers knapp vor der posterioren Wurzel des Außenmeniskus [1, 8].

Neuere anatomische Arbeiten von Śmigielski und Siebold sehen jedoch die knöcherne tibiale Insertion eher als „entenfußartig“, wobei der bandförmige funktionelle Hauptansatz c‑förmig von der lateralen Begrenzung des medialen Interkondylarhöckers bis zum anterioren Anteil der Außenmeniskusvorderhornwurzel reicht. Eine Unterscheidung zwischen einem anterolateralen und posteromedialen Bündel konnte hier nicht gefunden werden.

Im Jahr 2015 zeigten Guenther et al., dass doch eine große Varianz bezüglich der **tibialen Insertion**

Tibialen Insertion

herrscht. Sie beschrieben 3 unterschiedliche Ausprägungen der tibialen Insertion: elliptisch, triangulär und c‑förmig [9].

Das VKB wird von 2 Schichten Synovialis umhüllt und liegt somit zwar intraartikulär, aber extrasynovial. Femoralseitig wird das vordere Kreuzband von Endästen der A. genu medialis versorgt, tibialseitig aus den Aa. inferiores medialis und lateralis genu. Es zeigte sich jedoch eine **avaskuläre Zone**

Avaskuläre Zone

in der Insertionsregion und im vorderen distalen Drittel des VKB. Dies mag eine Erklärung für eine schlechte Heilungspotenz einer VKB-Ruptur sein [4, 10].

Die **Innervation**

Innervation

des VKB besteht aus einem ausgedehnten Netzwerk von **Rezeptoren**

Rezeptoren

wie dem Golgi-Sehnenorgan (Typ-III-Rezeptoren) sowie Ruffini- und Pacini-Körperchen (Typ-II-Rezeptoren), aber auch frei endenden Nervenfasern. Diese spielen insbesondere in der Propriozeption des Kniegelenkes eine wichtige Rolle. Zudem scheinen diese Rezeptoren auch über eine neuromuskuläre Koppelung mit der agonistischen ischiokruralen Muskulatur in Verbindung zu stehen.

### Merke

Das vordere Kreuzband wird funktionell in ein **anteromediales**

Anteromedial

und ein **posterolaterales**

Posterolateral

Bündel unterteilt.

## Biomechanik

Im Kniegelenk ist das VKB für die Begrenzung der anterioren tibialen **Translation**

Translation

verantwortlich. Während der Bewegungsphase des Kniegelenkes kommt es zu einer Anspannung unterschiedlicher Anteile des VKBs. Ältere in-vitro-Arbeiten an Leichenkniegelenken zeigten, dass in Streckstellung v. a. der posterolaterale Kreuzbandanteil, in 30° Beugestellung der intermediäre und in hoher Flexion der anteromediale Anteil gespannt sind. Jedoch konnte mittels Längenmessungen in der Magnetresonanztomographie (MRT) ein nahezu isometrisches Verhalten der anteromedialen Anteile nachgewiesen werden [11, 12]. Zudem hat das VKB auch eine Auswirkung auf die **Rotationsstabilität**

Rotationsstabilität,

indem es insbesondere in Streckstellung das Femur in eine geringe Innenrotationsstellung bringt. Eine Insuffizienz des VKB führt somit zu einer anterolateralen Subluxationsstellung der Tibia zum Femur und zu einer Verschiebung der Rotationsachse von zentral nach medial [13, 14].

Durch diese Kinematikveränderung kommt es zum einen zu einer nach posterior verlagerten Druckbelastung im medialen und lateralen Kompartiment des Kniegelenkes. Zum anderen führt die fehlende Fixierung des Femurs in geringer Innenrotation in Streckstellung zu einer vermehrten tibialen Innenrotation und Subluxation. Diese Mechanismen werden verantwortlich gemacht für die typische posteromediale Arthrose in VKB-insuffizienten Kniegelenken [15, 16].

## Klinische Untersuchung

Zunächst sollte im Rahmen der **Anamnese**

Anamnese

der genaue Verletzungsmechanismus erfragt werden. Hier können schon erste Hinweise auf mögliche Verletzungsarten erfolgen.

Das Vorliegen eines **Kniegelenkergusses**

Kniegelenkerguss

sollte palpatorisch oder auch sonographisch überprüft werden, da ein Erguss nach stattgehabtem Kniegelenkdistorsionstrauma ein Indikator für ein Kniebinnentrauma ist. Bei Beschwerden aufgrund des Kniegelenkergusses kann unter sterilen Kautelen eine Punktion erfolgen. Die Art des Ergusses (z. B. blutig oder serös, „Fettaugen“) lässt auch Rückschlüsse auf die Verletzung zu.

Bei der weiteren klinischen Untersuchung des Kniegelenkes sollte insbesondere auf die **kreuzbandtypischen Pathologien**

Kreuzbandtypischen Pathologien

geachtet werden [17]. Eine Ruptur des VKB führt zu einer vermehrten anterioren tibialen Translation, aber auch zu einer anterolateralen Subluxationsstellung der Tibia. Diese beiden Phänomene sind Grundlage für die klinischen Tests.

Das Ausmaß der anterioren Tibiatranslation wird durch den **Lachman-Test**

Lachman-Test

und den **vorderen Schubladentest**

Vorderer Schubladentest

bestimmt.

Der Lachman-Test und der vordere Schubladentest sind nur dann als positiv zu werten, wenn zuvor eine hintere Kreuzbandinsuffizienz ausgeschlossen wurde, da eine Instabilität des hinteren Kreuzbandes (HKB) eine vermehrte anteriore tibiale Translation vortäuschen kann.

Ein eingeschlagener Korbhenkelriss, freie Gelenkkörper oder auch arthrotische Veränderungen können jedoch umgekehrt trotz gerissenen VKB zu einem verkürzten vorderen Weg mit hartem Anschlag führen, also zu einem falsch negativen Ergebnis.

Vorteile des Lachman-Tests im Vergleich zum vorderen Schubladentest sind, dass ein vorliegender Hämarthros nur geringere Auswirkungen auf den Test hat und er aufgrund der geringeren Beugestellung schmerzärmer durchführbar ist. Auch kommt es beim Lachman-Test zu keiner (potentiellen) Interferenz der anterioren Tibiatranslation durch das mediale Seitenband oder das Meniskushinterhorn.

Die **Pivot-Shift-Tests**

Pivot-Shift-Tests

nützten das anterolaterale Subluxationsphänomen der Tibia im Vergleich zum Femur bei VKB-suffizienten Kniegelenken aus. Der bekannteste und wohl am häufigsten durchgeführte Pivot-Shift-Test ist der Test nach Galway und McIntosh [18]. Dieser Test ist jedoch sehr untersucherabhängig und zeigt aus diesem Grund in der Literatur eine große Varianz in Sensitivität und Spezifität.

## Instrumentelle Untersuchung

Da die klinische Untersuchung und Bewertung der anterioren Tibiatranslation sehr abhängig vom jeweiligen Untersucher, aber auch von der Konstitution des Patienten ist, herrscht eine große Varianz in der Beurteilung einer vorliegenden vorderen Schublade. Um hier eine exaktere Einschätzung zu erhalten, wurden im Laufe der Jahre verschiedene **technische Messinstrumente**

Messinstrumente

entwickelt. In Studien konnte ein positiver Effekt der instrumentellen Stabilitätsprüfung auf die Beurteilung des Ausmaßes der vorderen Schublade nachgewiesen werden. Somit kann die instrumentelle Messung, insbesondere im Seitenvergleich, zur Beurteilung der vorderen Kreuzbandinsuffizienz, aber auch in der Therapieentscheidung, ob konservativ oder operativ verfahren werden soll, helfen. Es konnte in Studien gezeigt werden, dass eine größere Seitendifferenz der vorderen Schublade eine operative Therapie eher wahrscheinlich macht und Patienten mit einer geringen Seitendifferenz eher einer konservativen Therapie zugeführt werden können [19, 20, 21].

## Bildgebung

### Konventionelles Röntgen

In der Akutversorgung von Knieverletzungen sollten zunächst konventionelle Röntgenaufnahmen des Kniegelenkes in 2 Ebenen (a.-p. und seitlich) durchgeführt werden. Hier können sich schon indirekte Zeichen einer VKB-Ruptur, wie z. B. die **Segond-Fraktur**

Segond-Fraktur,

zeigen. Dies ist eine Avulsionsfraktur des anterolateralen Ligamentes an der anterolateralen Tibia und meist vergesellschaftet mit einer VKB-Ruptur. Zudem können knöcherne vordere Kreuzbandausrisse, häufiger bei Kindern, erkannt werden.

Im **Revisionsfall**

Revisionsfall

sollte bei Verdacht auf einen vermehrten **tibialen Slope**

**Figure 1:**
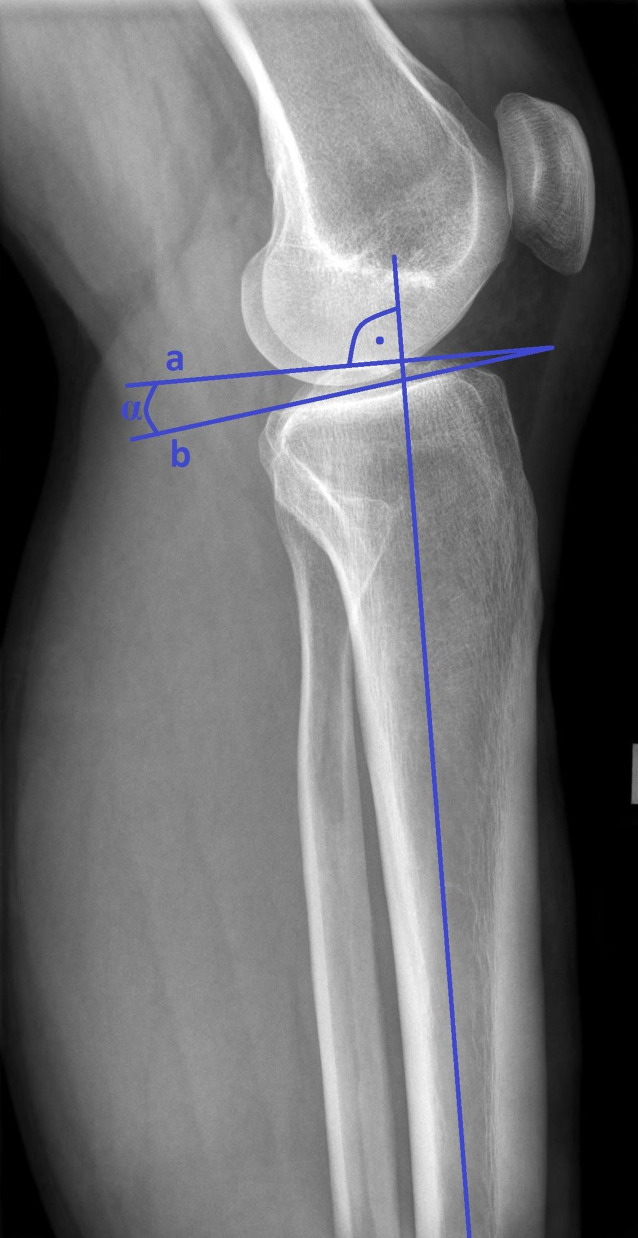

Tibialer Slope

(Neigung des Tibiaplateaus in Bezug auf die Tibiaschaftachse) eine seitliche Röntgenaufnahme des Kniegelenkes mit langer Darstellung des Unterschenkels angefertigt werden. Es werden verschiedene Messmethoden beschrieben. Zumeist wird der Winkel zwischen der Neigung des Tibiaplateaus und der anatomischen Schaftachse gemessen (Abb. 1). Die physiologische Dorsalneigung des Tibiaplateaus beträgt zwischen 7 und 13°. Ein vermehrter Slope erhöht die Last auf das vordere Kreuzband und kann ein Versagen der vorderen Kreuzbandersatzbandplastik begünstigen [22]. Somit kann bei Revisionen ab einem pathologischen Slope von über 12° eine Korrektur des tibialen Slope durch eine Osteotomie in Betracht gezogen werden. Hierdurch kann das Risiko von Rerupturen deutlich verringert werden. Eine alleinige Slope-Korrektur kann zwar in manchen Fällen die Stabilität des Kniegelenks positiv beeinflussen, doch eine Kombination mit einer VKB-Ersatzbandplastik zeigt deutlich bessere Ergebnisse [23].

Bei klinischem Verdacht auf eine höhergradige Varus- oder Valgusfehlstellung sowie im Revisionsfall sollte eine **Ganzbeinstandaufnahme**

Ganzbeinstandaufnahme

durchgeführt werden. Ein Varusmalalignment kann im Weiteren zu einer Elongation der lateralen Bandstrukturen (Double-Varus) und so zu einer vermehrten lateralen Gelenköffnung bei Belastung führen (Varus-Thrust). In der Folge kommt es zu einer Auslockerung der posterolateralen Strukturen (Tripple-Varus), und es entsteht dann unter Belastung ein Hyperextensions-Varus-Thrust. Ein **Varus-Thrust**

Varus-Thrust

führt zu einer bis um 218 % gesteigerten Belastung des vorderen Kreuzbandes und ist somit ein hoher Risikofaktor für ein Versagen der Bandplastik. Eine additive Umstellungsosteotomie ist in diesen Fällen eine effektive Behandlungsmethode [23].

**Gehaltene Aufnahmen**

Gehaltene Aufnahmen

spielen in der Diagnostik der VKB-Verletzung nur eine untergeordnete Rolle. Jedoch sollten bei Verdacht auf eine hintere Kreuzbandverletzung/-insuffizienz gehaltene Aufnahmen im Seitenvergleich in hinterer und vorderer Schublade zur Abklärung der HKB-Instabilität durchgeführt werden.

### Magnetresonanztomographie (MRT)

**Figure 2:**
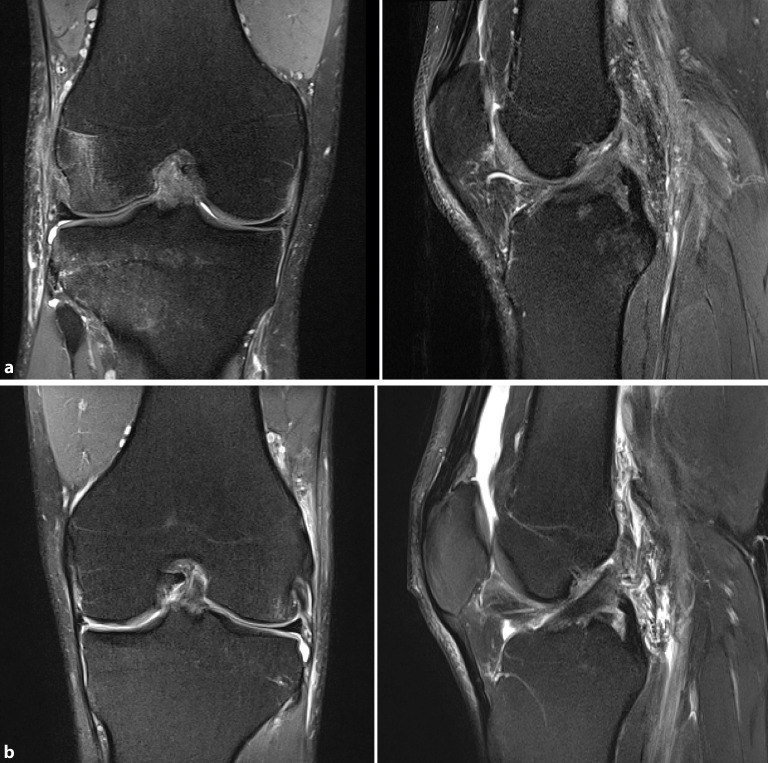

Die MRT-Untersuchung ist zur Abklärung einer vorderen Kreuzbandverletzung die Methode der Wahl (Abb. 2). **Direkte Zeichen**

Direkte Zeichen

einer VKB-Ruptur sind im Wesentlichen Kontinuitätsunterbrechungen, fehlender Nachweis des Bandes in anatomischer Position, wellige Kontur des VKB, Verlagerung des tibialen oder femoralen Bandansatzes bzw. Auftreibung oder diffuse Signalstörung des VKB. Neben den direkten Zeichen einer Rissbildung können auch **indirekte Hinweise**

Indirekte Hinweise

auf eine VKB-Ruptur in der MRT nachgewiesen werden. So ist ein „bone bruise“ im Bereich des posterolateralen Tibiaplateaus und korrespondierend im Bereich des Sulcus terminalis des lateralen Femurkondylus ein pathognomonisches Zeichen für eine vordere Kreuzbandruptur, aber auch ein Hinweis auf eine erhöhte Rotationsfehlstellung zum Zeitpunkt des Unfalls. Des Weiteren können in der MRT **Begleitverletzungen**

Begleitverletzungen

wie Knorpelverletzungen, Meniskusrisse und weitere Bandverletzungen abgeklärt werden.

#### Merke

Die **Magnetresonanztomographie (MRT)**

Magnetresonanztomographie (MRT)

ist die Untersuchungsmethode der Wahl zur Diagnostik vorderer Kreuzbandverletzungen.

### Computertomographie (CT)

**Figure 3:**
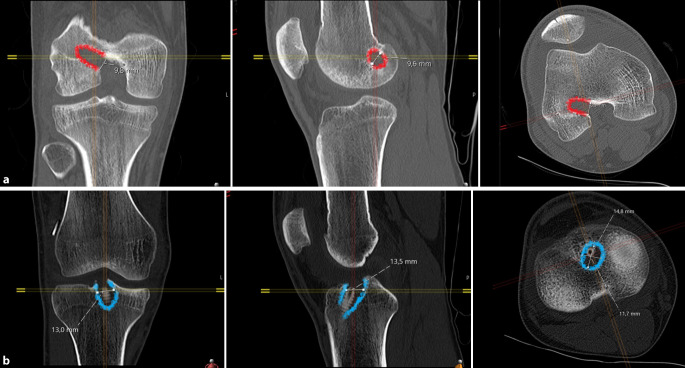

Eine native Computertomographie(CT)-Abklärung kann bei begleitenden Frakturen sinnvoll sein. Im Revisionsfall sollte eine CT zur Beurteilung der Bohrkanallage und Bohrkanalweite durchgeführt werden (Abb. 3). Die **Evaluierung der Bohrkanäle**

Evaluierung der Bohrkanäle

ist von entscheidender Bedeutung, ob eine ein- oder zweizeitige VKB-Revisionsersatzbandplastik mit vorheriger Bohrkanalauffüllung durchgeführt werden muss.

Eine **CT-Arthrographie**

CT-Arthrographie

(Arthro-CT) kann zur Diagnostik herangezogen werden, wenn eine MRT-Untersuchung aus medizinischen Gründen (z. B. kardiologische Implantate) nicht möglich ist. Im Gegensatz zur MRT bestehen hier die Notwendigkeit einer intraartikulären Kontrastmittelapplikation und zusätzlich die CT-bedingte Strahlenbelastung. Bei ähnlicher Spezifität ist die Sensitivität jedoch im Vergleich zur MRT geringer. Somit ist die CT-Arthrographie kein Mittel der ersten Wahl zur primären Diagnostik.

## Therapiealgorithmus

### Akute vordere Kreuzbandruptur

VKB-Rupturen können konservativ oder operativ behandelt werden. Eindeutige, klare, evidenzbasierte Empfehlungen zur Versorgung einer vorderen Kreuzbandruptur liegen zum aktuellen Zeitpunkt nicht vor. Für den Therapieentscheid existieren jedoch Kriterien, die für oder gegen eine operative Therapie sprechen. Hier spielen das Patientenalter, das Aktivitätslevel, die Kniegelenkstabilität, die Begleitverletzungen und das Operationsrisiko eine Rolle. Der Zeitpunkt der Versorgung richtet sich auch nach den Begleitverletzungen. Ein **Behandlungsalgorithmus**

**Figure 4:**
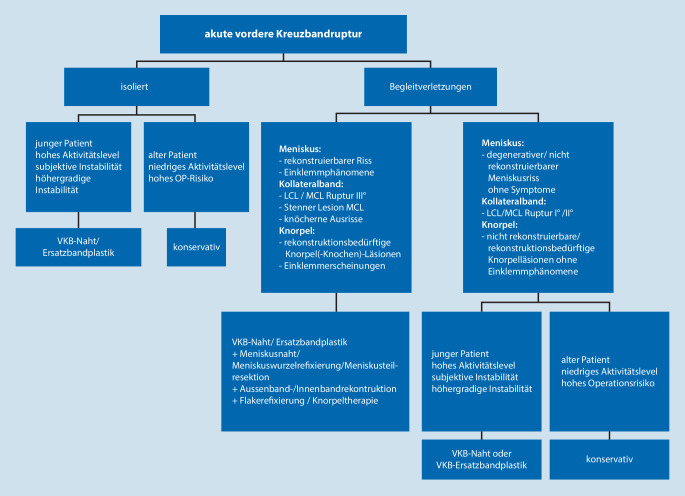

Behandlungsalgorithmus

ist in Abb. 4 dargestellt.

Eine **isolierte VKB-Ruptur**

Isolierte VKB-Ruptur

kann entweder innerhalb der ersten 48 h nach dem Unfall oder nach Abklang der akuten traumatischen Entzündungsphase, meist nach ca. 4 bis 6 Wochen, operativ versorgt werden. Das Kniegelenk sollte zum Zeitpunkt der Ersatzbandplastik abgeschwollen, reizlos und nicht mehr schmerzhaft sein. Zudem sollten eine Beugung von über 90° und eine nahezu komplette Streckung erreicht werden.

Ist ein vorderer **Kreuzbanderhalt**

Kreuzbanderhalt

geplant, so wird eine Operation innerhalb von 2 bis 3 Wochen nach dem Trauma empfohlen.

Relevante Begleitverletzungen benötigen in der Regel ebenfalls ein zeitnahes Vorgehen innerhalb von 2 bis 3 Wochen. In diesen Fällen muss individuell abgewogen werden, ob eine simultane VKB-Rekonstruktion durchgeführt wird. Nach wie vor wird über eine erhöhte **Arthrofibroserate**

Arthrofibroserate

nach einer VKB-Ersatzbandplastik während der posttraumatischen Entzündungsphase diskutiert. Jedoch konnte auch gezeigt werden, dass eine einzeitige Meniskusnaht in Kombination mit einer VKB-Rekonstruktion bessere Ergebnisse liefert als ein zweizeitiges Vorgehen.

### Chronische vordere Kreuzbandinsuffizienz

Eine chronische vordere Kreuzbandinsuffizienz tritt zum einen nach fehlgeschlagener konservativer oder ausgebliebender Therapie und zum anderen nach fehlgeschlagener VKB-Rekonstruktion auf.

Insbesondere bei einer **Rezidivinstabilität**

Rezidivinstabilität

nach vorderer Kreuzbandoperation muss eine ausgiebige **Fehleranalyse**

Fehleranalyse

betrieben werden und diese in die Therapie mit einbezogen werden.

**Figure 5:**
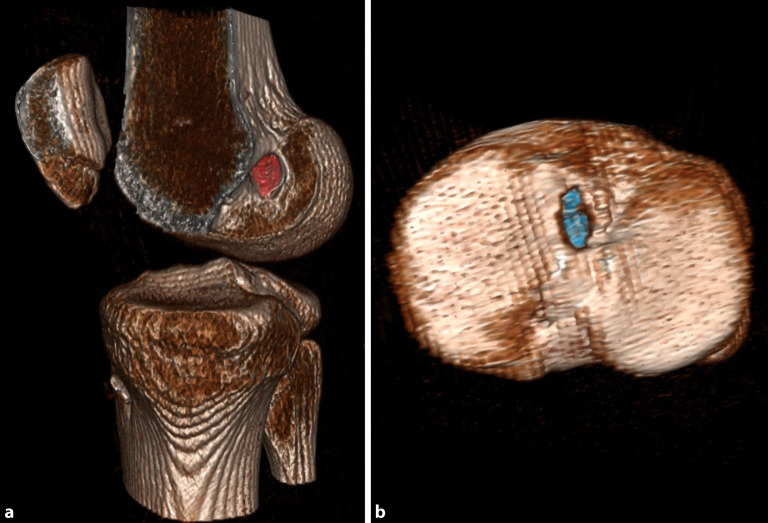

Abzuklären sind insbesondere:

- zusätzliche Begleitinstabilitäten (anteromediale/anterolaterale Instabilität, HKB-Insuffizienz) durch klinische Untersuchung, MRT, gehaltene Röntgenaufnahmen mit vorderer und hinterer Schublade,
- höhergradige Beinachsenfehlstellungen durch Ganzbeinstandröntgen a.-p.,
- höhergradiger tibialer Slope durch Röntgen des Kniegelenkes mit langem Unterschenkel seitlich,
- Fehlplatzierung der Bohrkanäle bzw. eine Bohrkanalerweiterung durch Computertomographie, ggf. mit 3‑D-Rekonstruktion (Abb. 5).

Zeigt sich in der **Analyse der Bohrkanäle**

Analyse der Bohrkanäle

eine teilweise fehlerhafte Tunnelplatzierung, sodass diese mit den neuen anatomischen Bohrkanälen konfluieren würden, oder zeigt sich eine übermäßige Bohrkanalerweiterung bei korrekter Bohrkanallage, so sollte ein zweizeitiges Vorgehen gewählt werden. In einem ersten Eingriff werden die Bohrkanäle mit autologem und/oder allogenem Knochenmaterial aufgefüllt. Nach der Konsolidierung ca. 3 bis 6 Monate postoperativ kann dann die **zweizeitige VKB-Revisionsersatzbandplastik**

Zweizeitige VKB-Revisionsersatzbandplastik

durchgeführt werden.

Auch bei der chronischen vorderen Instabilität nach erfolgloser konservativer Behandlung sollte auf die oben genannten ungünstigen prognostischen Faktoren geachtet werden.

#### Cave

Bei **chronischen**

Chronisch

oder **rezidivierenden**

rezidivierenden

vorderen Kreuzbandverletzungen ist eine ausgiebige **Fehleranalyse**

Fehleranalyse

durchzuführen.

### Begleitverletzungen

#### Meniskusrampenläsion

Als Meniskusrampe wird die fächerförmige meniskotibiale Verbindung des Innenmeniskushinterhorns beschrieben. Diese stabilisiert den Innenmeniskus in a.-p.-Richtung. Eine Verletzung führt gemäß biomechanischen Untersuchungen somit zu einer vermehrten **anteroposterioren Knielaxizität**

Anteroposteriore Knielaxizität.

Mittels MRT lässt sich derzeit eine Meniskusrampenläsion nicht suffizient darstellen. So ist der diagnostische Goldstandard aktuell die direkte **arthroskopische Untersuchung**

Arthroskopische Untersuchung

durch die Visualisierung der Meniskusrampe im posteromedialen Kompartiment, ggf. auch über ein posteromediales Portal. Da die meniskotibialen Bänder von der Synovialmembran und Gelenkkapsel bedeckt sind, kann die Beurteilung erschwert sein. Bei vorliegender Meniskusrampenläsion wird die direkte Naht über ein posteromediales Portal empfohlen. Gemäß aktuellen Untersuchungen treten Meniskusrampenläsionen in bis zu 20–30 % bei vorderen Kreuzbandrupturen auf. Somit sollte bei jeder Kreuzbandversorgung auch ein Blick auf die Meniskusrampe geworfen werden [24, 25].

#### Mediale Kollateralbandläsion

Bei Läsionen des medialen Kollateralbandes (MCL) kommt es unter Außenrotation der Tibia zu einer vorderen Subluxation des medialen Plateaus. Anhand von biomechanischen Studien konnte die Relevanz der einzelnen **Innenbandstrukturen**

Innenbandstrukturen

(tiefes Innenband – dMCL, oberflächliches Innenband – sMCL und hinteres Schrägband – POL) untersucht und daran die anteromediale Instabilität klassifiziert werden [26]. Spätestens bei Grad-III-Läsionen sollte eine mediale Rekonstruktion begleitend zur vorderen Kreuzbandrekonstruktion erfolgen.

#### Laterale Kollateralbandläsion

Laterale Kollateralband(LCL)-Verletzungen treten meist in Kombination mit Verletzung der zentralen Bandstrukturen auf. Partielle Rissbildungen ohne höhergradige Instabilität können konservativ behandelt werden. Jedoch sollte bei Komplettrupturen oder **höhergradigen Instabilitäten**

Höhergradige Instabilitäten

eine Rekonstruktion begleitend zur vorderen Kreuzbandrekonstruktion erfolgen. Bei einer **chronischen Instabilität**

Chronische Instabilität

muss diese ebenfalls mit adressiert werden. Hier stehen verschiedene operative Verfahren, wie z. B. die Stabilisierungsoperationen in der Larson-Technik oder der La Prade-Technik oder die Umlenkungs‑/Versetzungsoperationen wie der Popliteusbypass oder die Bizepstenodese nach Clancy, zur Verfügung.

#### „Unhappy triad/tetrad“

Die **Kombinationsverletzung**

Kombinationsverletzung

von vorderem Kreuzband, medialem Kollateralband und Innenmeniskus wird als „unhappy triad“ bezeichnet [27]. Neuere Studien sprechen mittlerweile von einer **Tetrade**

Tetrade

anstelle einer Triade, da in den meisten Fällen auch eine Läsion des anterolateralen Komplexes nachgewiesen werden kann [28]. Insbesondere in Fällen von komplexerer Instabilität scheint neben der Rekonstruktion des vorderen Kreuzbandes eine zusätzliche anterolaterale Stabilisierung sinnvoll zu sein [29, 30].

### Transplantatwahl

Für die Wahl der Transplantate spielen neben deren Vorhandensein die berufliche wie sportliche Belastung des Patienten und die Begleitverletzungen eine Rolle. So sollte z. B. bei knieenden Berufen wie Fliesenleger auf eine Entnahme eines Patellasehnentransplantates verzichtet werden.

In Tab. 1 sind die Vor- und Nachteile der jeweiligen Transplantate aufgelistet:

**Table 1:** 

	Autogene Transplantate (ipsi- oder kontralateral)			Allogene Transplantate
	*Hamstringsehnen*	*Quadricepssehne*	*Patellasehne*	*Hamstringsehnen*
	Semitendinosussehne			*Quadricepssehne*
	Gracilissehne			*Patellasehne* *Achillessehne*
Vorteile	Einfache und schnelle Sehnenentnahme	Variable Transplantatlänge und -dicke	Schnellere Transplantateinheilung	Sehnenentnahme entfällt
	Geringe Entnahmemorbidität	Möglichkeit der Entnahme mit und ohne Knochenblock	Variierbare Transplantatbreite	Keine Entnahmemorbidität
	Hohe initiale Reißfestigkeit	Schwächung des wichtigsten Antagonisten des VKB	Schwächung des wichtigsten Antagonisten des VKB	Variable Transplantatlänge und -dicke
	Schonung des patellofemoralen Komplexes	Geringere Entnahmemorbidität und peripatellare Schmerzen als bei der Patellasehne	–	Möglichkeit der Verwendung mit und ohne Knochenblock
	Variable Transplantatlänge und -dicke	–	–	Keine Schwächung der Agonisten und Antagonisten
	Geringeres Risiko eines Zyklopssyndroms	–	–	–
Nachteile	Schwächung der Agonisten des VKB	Erhöhte postoperative Atrophie des M. quadriceps mit eingeschränkter Kniestreckung	Störung des patellofemoralen Gelenkes → patellofemorales Schmerzsyndrom	Verfügbarkeit in Deutschland erschwert
	Verstärkung einer medialen Knieinstabilität	Patellafraktur/Quadricepssehnenruptur (sehr selten)	Patellafraktur	Haftungsrisiko
	Längere Knocheneinheilung	–	Vorgegebene Transplantatlänge	–
	(Passagere) Reduktion der Beugekraft (geringer bei alleiniger Entnahme der Semitendinosussehne)	–	Entnahmedefekte im Bereich der Patella und der Tuberositas → Scherzen beim Hinknien	–
	Hyp‑/Dysästhesie durch Verletzung des Ramus infrapatellaris des N. saphenus	–	Langer Hautschnitt	–

Am häufigsten werden im Primärfall die **Hamstringsehnen**

Hamstringsehnen

verwendet. Jedoch erlebt die **Quadricepssehne**

Quadricepssehne

in den letzten Jahren eine Renaissance und findet insbesondere im Revisionsfall, aber auch bei zusätzlich medialer Knieinstabilität immer mehr Beliebtheit. Situativ bedingt, z. B. bei einer Revisions-VKB-Ersatzbandplastik, kann die Sehnenentnahme auch von der Gegenseite erfolgen.

Die Verwendung von **allogenen Transplantaten**

Allogenen Transplantaten

ist in Deutschland weit weniger verbreitet als im angloamerikanischen Raum. Ein Grund hierfür ist die komplizierte rechtliche Situation in Deutschland. Somit finden aktuell Allografts v. a. bei komplexen Revisionsfällen oder bei Multiligamentverletzungen Anwendung.

Durch Einlegen des Transplantates in einer **Vancomycin-Lösung**

Vancomycin-Lösung

vor der Implantation wird das Infektionsrisiko signifikant verringert [31].

#### Merke

Am häufigsten werden **Hamstringsehnen**

Hamstringsehnen

als Transplantat verwendet.

### Transplantatverankerung

#### Gelenknahe Fixation

Eine verbreitete Verankerungstechnik ist die gelenknahe Fixation mit **Interferenzschrauben**

Interferenzschrauben.

Hier wird mittels nichtresorbierbarer oder resorbierbarer Schrauben das Transplantat auf Gelenkniveau fixiert. Dadurch verringert sich die effektive Transplantatlänge auf die intraartikuläre Strecke und beträgt nur 2–3 cm. Dies erhöht im Vergleich zu den gelenkfernen Fixationstechniken die Steifigkeit des Transplantates. Des Weiteren ist die Gefahr einer Bohrkanalweitung geringer als bei gelenkferner Fixierung. Insbesondere bei Verwendung von reinen Sehnentransplantaten scheint sich durch die gelenknahe Verankerung schneller eine neue direkte Bandinsertion im Rahmen der Einheilung auszubilden. Jedoch führt die gelenknahe Fixation auch zu einer verminderten Kontaktfläche zwischen dem Transplantat und der Bohrkanalwand, was somit die knöcherne Einheilung stören kann.

#### Gelenkferne Fixation

Bei der gelenkfernen Fixation wird das Transplantat über eingelegte bzw. eingenähte Einzugsfäden außerhalb des Gelenkes fixiert. Dies kann über (Knoten-)**Plättchen**

Plättchen

oder **Schrauben**

Schrauben

erfolgen, gegen welche die Fäden meist auf der Kortikalis befestigt werden. Vorteile dieser Methode sind unter anderem die einfache Platzierung der Implantate und die hohe initiale Verankerungsfestigkeit. Insbesondere bei Verwendung von den Hamstringsehnen besteht durch verschiedene Präparationstechniken eine hohe Variabilität der Transplantatdicke. Durch diese Techniken ist zumeist die Semitendinosussehne ausreichend, sodass nur in Ausnahmefällen die Gracilissehne mit entnommen werden muss. Dies hat eine deutlich geringere Entnahmemorbidität zur Folge. Jedoch kann es zu einer erhöhten **Transplantatmobilität**

Transplantatmobilität

(Bungee‑/Scheibenwischereffekt) im Bohrkanal kommen mit den Folgen einer Tunnelweitung und einer verringerten Knocheneinheilung. Eine Vereinfachung der Handhabung konnte durch neuere justierbare Plättchen-Schlaufen-Systeme erreicht werden.

#### Implantatfreie Fixierung

Implantatfreie Verankerungstechniken basieren auf einer **Press-Fit-Verankerung**

Press-Fit-Verankerung.

Initial entwickelt für Bone-Tendon-Bone-Patellasehnentransplantate, gibt es auch Operationstechniken, die eine Press-Fit-Verankerung mit den Hamstringsehnen ermöglichen. Vorteile sind die Implantatfreiheit im Bohrkanal bei gleichzeitiger gelenknaher Fixierung und die geringeren Kosten. Nachteilig sind jedoch die teils aufwendigere Präparation und Operationstechnik. Zudem ist bei Verwendung der Hamstringsehnen eine höhere Transplantatlänge notwendig, wodurch meist die Gracilissehne mit entnommen werden muss.

## Operationstechniken bei Ersatzbandplastik

### Transtibiale Technik

In der transtibialen Technik erfolgt die Anlage des femoralen Bohrkanals über den tibialen Bohrkanal. Das femorale Zielgerät wird über den zuvor angelegten tibialen Tunnel eingebracht, und hierüber erfolgt auch die Bohrung des femoralen Kanals. Hierbei kommt es gehäuft zu einer nichtanatomischen Platzierung des femoralen Bohrkanals, zu weit anterior oder in „High-Noon“-Position. Dies führt zu einer verminderten Rotations- und a.-p.-Stabilität. Insgesamt zeigen sich in der Literatur schlechtere biomechanische und klinische Ergebnisse als bei einer femoralen Tunnelanlage über ein anteromediales Portal, sodass aus heutiger Sicht eine transtibiale Bohrtechnik nicht zu empfehlen ist [32, 33].

### Anteromediale Portaltechnik

Hier wird der femorale Bohrkanal über ein anteromediales **Arthroskopieportal**

Arthroskopieportal

angelegt, wodurch eine verbesserte anatomische Positionierung des femoralen Bohrkanals ermöglicht wird. Die anatomische Positionierung des femoralen Bohrkanals in dieser Technik ist jedoch nicht trivial, insbesondere da zur Tunnelanlage höhere Beugegrade (ca. 110–120° Flexion) notwendig sind. Dies führt zu einer schlechteren Einsicht der lateralen Notchwand, wodurch die regelrechte Zielgerätpositionierung erschwert wird. Von Vorteil ist jedoch die freie, unabhängig voneinander positionierbare Anlage des tibialen und femoralen Bohrkanals. Hierdurch kann ein anatomischer Verlauf der Ersatzbandplastik erreicht werden. Ein individuelles Anpassen des femoralen und tibialen Bohrkanals an den Transplantatdurchmesser ermöglicht zudem eine Press-Fit-Lage des Transplantates im Tunnel.

#### Merke

Die **anteromediale Portaltechnik**

Anteromediale Portaltechnik

ist die verbreitetste Operationstechnik zum vorderen Kreuzbandersatz.

### Outside-in-Technik/All-inside-Technik

In dieser Technik erfolgt die Anlage des femoralen **Zielbohrdrahtes**

Zielbohrdrahtes

analog zum tibialen Bohrkanal von extraartikulär nach intraartikulär. Der Vorteil dieser Technik ist die freie Positionierung des femoralen Bohrkanals in 90° Flexion. Da hier keine Flexion über 110° wie bei der anteromedialen Technik notwendig ist, ist die Visualisierung der lateralen Notchwand meist besser. Durch die **freie Positionierung**

freie Positionierung

besteht zudem die Möglichkeit einer exakten anatomischen femoralen Bohrkanalanlage. Durch spezielle retrograde Bohrer besteht die Möglichkeit einer knochensparenderen Bohrkanalanlage im Sinne von **Sacklöchern**

Sacklöchern.

**Figure 6:**
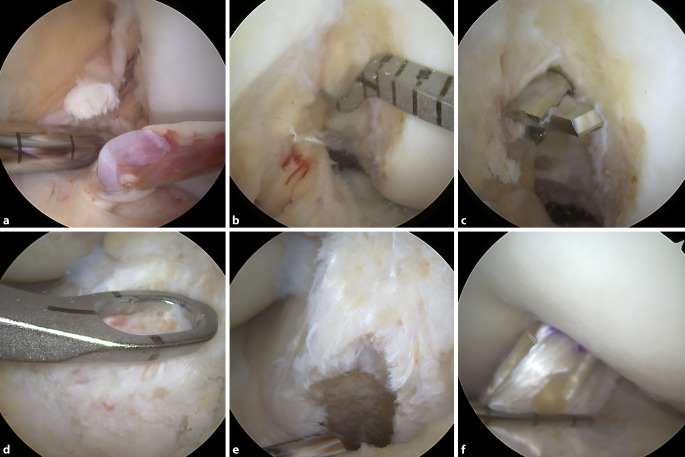

Im Vergleich zur anteromedialen Portaltechnik kann ein solches Sackloch hier auch tibial angelegt werden. Vorteil dieser All-inside-Technik (Abb. 6) ist, dass sie meist schon ab einer Transplantatlänge von 60 mm durchführbar ist, wodurch in der Regel die Semitendinosussehne ausreicht und die Gracilissehne belassen werden kann. Jedoch bringt diese All-inside Technik auch ein paar Besonderheiten mit sich. So muss die Transplantatlänge auf die Bohrkanallänge abgestimmt sein, da ein zu langes Transplantat bei zu kurzen Bohrkanälen zu einer laxen Bandplastik führen würde. Auch ist das retrograde Einziehen des Transplantates über das Arthroskopieportal in den tibialen Bohrkanal nicht ganz einfach. Bei dieser Technik kann ausschließlich eine gelenkferne Fixierung durchgeführt werden.

### Naht/Refixierung

Lange Zeit spielte die VKB-Refixierung oder Naht nur eine untergeordnete Rolle aufgrund schlechter Ergebnisse in der Vergangenheit. Durch modernere arthroskopische Techniken und Instrumente, aber auch durch ein verbessertes Verständnis, welche Kreuzbandrupturen erhaltungsfähig sind, erlebt der **vordere Kreuzbanderhalt**

vordere Kreuzbanderhalt

aktuell eine Renaissance. Es scheinen sich frische femorale oder tibiale Avulsionsverletzungen mit guter Stumpfqualität gut für den Erhalt zu eignen, wohingegen intraligamentäre Bandrupturen oder eine schlechte Stumpfqualität Kontraindikationen sind. Zudem sollte die Versorgung zeitnah innerhalb von 2 bis 3 Wochen nach dem Unfall erfolgen.

Bei den dynamischen Stabilisierungssystemen wird ein femoralseitig fixierter Polyethylenfaden im VKB-Verlauf tibialseitig ausgeleitet und über einem Monoblock mit Federmechanismus befestigt. Hierdurch wird eine dynamische Schienung des vorderen Kreuzbandes erreicht mit dem Ziel einer besseren Ausheilung der Naht. Es konnten mit dieser Technik vergleichbare funktionelle Ergebnisse wie bei einer VKB-Rekonstruktion erreicht werden bei jedoch höheren Rerupturraten.

**Figure 7:**
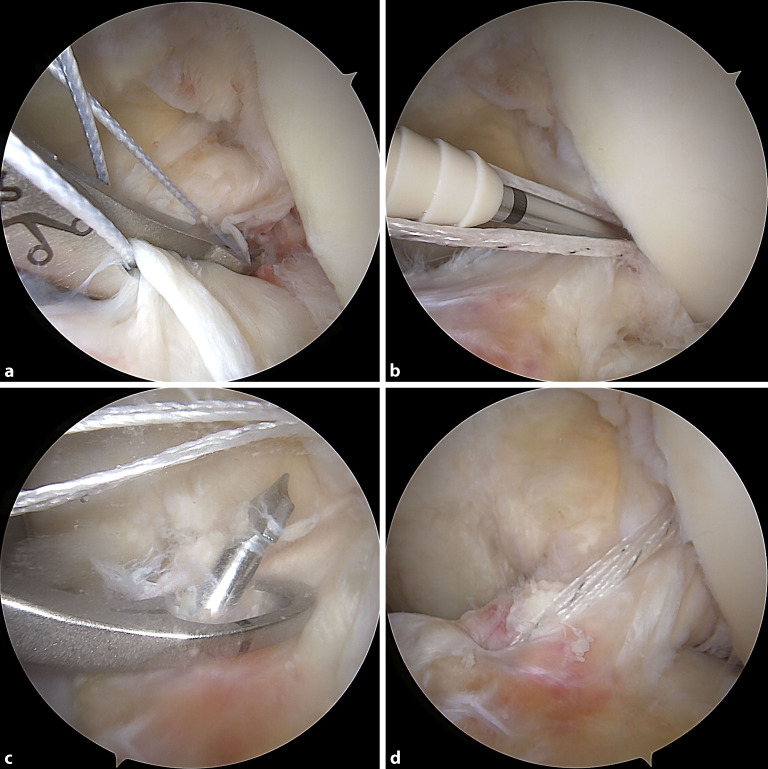

Das sog. Ligament-Bracing hingegen bewirkt eine statische Unterstützung des genähten oder refixierten vorderen Kreuzbandes. Hierbei wird ein (doppelt gelegtes) geflochtenes Fadenbandmaterial parallel zum vorderen Kreuzband als **interne Schienung**

interne Schienung

(„internal brace“) eingezogen. Durch das „internal bracing“ soll ein passagerer mechanischer Schutz der vorderen Kreuzbandnaht bis zu deren sicheren Ausheilung gewährleistet werden (Abb. 7).

Die **Naht**

Naht

bzw. **Reinsertion**

Reinsertion

einer femoralen bzw. knöchernen tibialen VKB-Ruptur kann auch isoliert ohne die oben genannten intraartikulären Schienungstechniken durchgeführt werden. Vorteile hier sind die kürzere und einfachere Operationstechnik, die geringeren Materialkosten sowie weniger intraartikuläres Fremdmaterial.

Eine solche Naht bzw. Reinsertion erfolgt zunächst durch Anschlingen des vorderen Kreuzbandstumpfes. Die Fixierung erfolgt femoralseitig entweder mit einem knotenlosen Fadenankersystem oder gelenkfern über ein (Knoten‑)Plättchen. Tibial wird stets eine gelenkferne Fixierung durchgeführt.

Ob und wann eine interne Schienung, dynamisch oder statisch, zusätzlich zur Naht notwendig ist, müssen weitere Studien noch zeigen.

### Stumpferhaltende Technik

Aufgrund neuerer histologischer Studien zum vorderen Kreuzband besteht der Trend, möglichst viel des rupturierten Stumpfes zu erhalten. Hierdurch erhofft man sich eine verbesserte **postoperative Propriozeption**

postoperative Propriozeption

und durch den Erhalt des Synovialschlauches eine **schnellere Vaskularisierung**

schnellere Vaskularisierung.

Eine erhöhte postoperative Zyklopsrate durch das Belassen von Stumpfanteilen, wie es früher befürchtet wurde, konnte bis dato nicht nachgewiesen werden. Eine Überlegenheit der stumpferhaltenden VKB-Rekonstruktion im Vergleich zur stumpfresezierenden Technik konnte zwar aktuell noch nicht eindeutig gezeigt werden, jedoch sehen wir den Erhalt von Mechano- und Dehnungsrezeptoren sowie von Synovialgewebe mit mesenchymalen Stammzellen durchaus sinnvoll. Zudem können durch das Belassen der femoralen und tibialen Stümpfe die patientenindividuellen anatomischen Gegebenheiten besser beachtet werden.

### Einzelbündel- vs. Doppelbündeltechnik

Infolge von anatomischen Studien, die eine Unterteilung des vorderen Kreuzbandes in ein anteromediales und eine posterolaterales Bündel feststellten, wurde die **Doppelbündeltechnik**

Doppelbündeltechnik

entwickelt. Ziel dieser isolierten Anlage beider Bündel war es, eine verbesserte anatomische und biomechanische Rekonstruktion zu erreichen. Zwar konnten mit der Doppelbündeltechnik in Kadaveruntersuchungen bessere biomechanische Ergebnisse erreicht werden, jedoch zeigten bislang klinische Studien keine eindeutige signifikante Überlegenheit der Doppelbündel- gegenüber der Einzelbündeltechnik [34]. Nachteile der Doppelbündeltechnik sind der vermehrte zeitliche Aufwand, die komplexere Operationstechnik, die erhöhten Implantatkosten und die erschwerten Bedingungen im Revisionsfall.

### Neue Techniken

Śmigielski konnte in neueren anatomischen Studien eine längliche femorale und eine c‑förmige tibiale Insertion sowie einen flachen Bandverlauf zeigen [3]. Die neue **Ribbon-VKB-Ersatzbandplastiktechnik**

Ribbon-VKB-Ersatzbandplastiktechnik

setzt diese anatomische Gegebenheit um. Es werden mittels spezieller Zielgeräte sowie spezieller Bohr- und Bougierungstechnik femoral ein länglicher Bohrkanal und tibial ein c‑förmiger Bohrkanal geschaffen. Inwieweit dadurch verbesserte klinische Ergebnisse erreicht werden können, muss erst noch durch Studien gezeigt werden.

### Zusätzliche anterolaterale Stabilisierung

Seit den Veröffentlichungen von Claes et al. 2013 erhält der **anterolaterale Bandkomplex**

anterolaterale Bandkomplex,

bestehend aus dem Tractus iliotibialis mit den Kaplan-Fasern, dem anterolateralen Ligament und der anterolateralen Kapsel, eine enorme Beachtung. Eine vermehrte anterolaterale Rotationsinstabilität zeigt sich klinisch in einem höhergradigen positiven Pivot-Shift-Test [35].

Wann eine zusätzliche anterolaterale Stabilisierung durchgeführt werden sollte, ist noch nicht endgültig geklärt. Die Empfehlungen sind jedoch aktuell bei höhergradigem positivem Pivot-Shift-Test, bei einer zusätzlich vorliegenden Segond-Fraktur, bei Rerupturen und bei Profi- oder Wettkampfsportlern mit pivotierenden Sportarten. Zudem kann eine anterolaterale Stabilisierung bei persistierender Rotationsinstabilität nach VKB-Plastik durchgeführt werden.

Bezüglich der Operationstechnik wird aktuell zwischen der nichtanatomischen extraartikulären Stabilisierung in der modifizierten Lemaire-Technik mittels Tractusstreifen und der anatomischen Rekonstruktion des anterolateralen Ligamentes mittels autologer Gracilissehne unterschieden. Ein eindeutiger Vorteil einer dieser Techniken konnte bislang nicht beschrieben werden. In ersten Untersuchungen scheint eine Kombination aus VKB-Ersatzbandplastik und anterolateraler Stabilisierung zu einer geringeren Rerupturrate und zu sehr guten, teils der isolierten VKB-Ersatzbandplastik überlegenen klinischen Ergebnissen zu führen [29, 30].

### Komplikationen

Neben allgemeinen Operationsrisiken sind für die VKB-Ersatzbandplastik im Wesentlichen folgende typische Komplikationen beschrieben:

- **Infektionen**
  Infektionen
  – Die Inzidenz wird je nach Quelle mit bis zu 2 % angegeben, wobei diese durch den Einsatz durch lokale Vancomycin-Applikation auf das Kreuzbandtransplantat deutlich reduziert werden können [36]. Ein stadienadaptiertes Therapieschema bei Infektionen wurde von Petersen et al. 2014 veröffentlich und kürzlich aktualisiert [37, 38].
- **Arthrofibrose**
  Arthrofibrose
  – Sie zählt ebenfalls zu den häufigeren Komplikationen. Sie ist primär bedingt durch chronische inflammatorische Gewebezunahme oder sekundär aufgrund von Transplantatfehlplatzierung, Notchimpingement oder Zyklopssyndromen.
- **Fehlplatzierung**
  Fehlplatzierung
  – Neben der Arthrofibrose kann eine Transplantatfehlplatzierung auch zu einer primär persistierenden Instabilität führen, wenn der anatomische Verlauf des VKB nicht nachempfunden wird.
- **Auslockerungen**
  Auslockerungen
  – Sie führen sekundär zu einer persistierenden Instabilität. Diese können durch Bohrkanalerweiterungen, Versagen der Fixation oder auch (Low-grade‑)Infekte entstehen.

## Konservative Therapie und Nachbehandlung

In aktuellen Analysen konnte die operative Kreuzbandersatzbandplastik einen Vorteil gegenüber der konservativen Therapie zeigen und wird insbesondere bei jungen, sportlich aktiven Patienten aktuell empfohlen. Jedoch können verschiedene Faktoren, wie z. B. Patientenalter oder -konstitution sowie mögliche Grund- oder Begleiterkrankungen, eine konservative Therapie bedingen. Die konservative Therapie ähnelt der Nachbehandlung einer operativen Therapie. Aktuell wird immer mehr von den rein zeitbasierten Nachbehandlungsschemata Abstand genommen und zu einer **kriterienbasierten Rehabilitation**

kriterienbasierten Rehabilitation

mit der Hilfe von verschiedenen Funktionstest bzw. Testbatterien gewechselt. Um die jeweils nächste Behandlungsphase zu erreichen, sollten objektive Parameter durch **Funktionstests**

Funktionstests

herangezogen werden. Mit diesen Return-to-activity/-sport/-play/-competition-Tests lässt sich so zum einen die Nachbehandlung individuell auf den Patienten und dessen jeweiligen Leistungsstand ausrichten und zum anderen das Risiko einer erneuten Verletzung verringern.

## Prävention

Vordere Kreuzbandverletzungen haben weitreichende Folgen für einen sportlich aktiven Menschen. Eine chronische vordere Kreuzbandinstabilität führt zu einer signifikanten Zunahme von relevanten Meniskus- und Knorpelschäden. Eine vordere Kreuzbandersatzbandplastik reduziert diese Folgeschäden und damit die Arthroseprogredienz des Kniegelenkes. Die Rate der Patienten, die auf ihr altes sportliches Niveau kommen, beträgt jedoch nur ca. 65 % bei Hobbysportlern und 83 % bei Eliteathleten [39, 40, 41]. Diese Daten verdeutlichen die Relevanz, präventiv tätig zu werden. So wurden im Laufe der letzten Jahre verschiedene **Präventionsprogramme**

Präventionsprogramme

entwickelt. Durch diese speziellen Programme können die Raten an vorderer Kreuzbandrupturen um 51 % reduziert werden [42].

## Fazit für die Praxis

- Vordere Kreuzbandverletzungen gehören zu den häufigsten Sportverletzungen in Deutschland.
- Die persistierende Instabilität am Kniegelenk führt vermehrt zu Folgeschäden von Menisken und Knorpel und somit zu einer Arthroseprogredienz.
- Die Therapie sollte in Abhängigkeit von Begleitverletzungen und insbesondere bei Revisionen in Abhängigkeit von Begleitpathomorphologien erfolgen.
- Durch aktuelle arthroskopische Operationsverfahren können gute Behandlungsergebnisse erzielt werden.
- Das aktuell geläufigste Verfahren zur Versorgung der vorderen Kreuzbandruptur ist die Ersatzplastik in anatomischer arthroskopischer Technik unter Verwendung eines freien mehrsträngigen autologen Sehnentransplantates aus der Pes-anserinus-Gruppe (Semitendinosus- oder Semitendinosus- und Gracilissehne).
- Die Verwendung von alternativen autologen Transplantaten wie Quadriceps‑/Patellasehne, aber auch die Naht des vorderen Kreuzbandes sind je nach Situation weitere Therapieoptionen.
- Für die aktuellen VKB-Nahttechniken fehlen bislang breit aufgestellte Daten für die klinischen Ergebnisse.

## CME-Fragebogen

Welche Bewegungsebenen werden durch das vordere Kreuzband am Kniegelenk stabilisiert?

Die posteriore Translation

Die mediolaterale Translation

Die posteriore Translation und die Rotation in Beugung

Die mediolaterale und anteriore Translation

Die anteriore Translation und die Rotation

Ein 23-jähriger Mann stellt sich nach einem Sturz beim Skifahren mit Schwellung, Schmerzen und subjektivem Instabilitätsgefühl in Ihrer Ambulanz vor. Die Beugung ist schmerzbedingt nur bis ca. 50°, die Streckung nahezu vollständig möglich. Im konventionellen Röntgen zeigen sich keine Hinweise auf eine Fraktur. Welche klinischen Untersuchungsmethoden sind zur Beurteilung des vorderen Kreuzbandes geeignet?

Schubladentest in Kombination mit Steinmann-Test

Lachman-Test in Kombination mit Pivot-Shift-Test

Steinmann-Zeichen I und II in Kombination mit Pivot-Shift-Test

Single- und Tripple-Leg-Hopp-Test

Untersuchung der Außenrotation in Bauchlage bei 30 und 90° Flexion

Was ist hinsichtlich der Bildgebung im Rahmen der Diagnostik des vorderen Kreuzbandes richtig?

Im konventionellen Röntgenbild in 2 Ebenen kann eine vordere Kreuzbandruptur mit hoher Sicherheit ausgeschlossen werden.

Die Magnetresonanztomographie (MRT) ist der Goldstandard zur radiologischen Beurteilung des Kreuzbandes und vieler Begleitverletzungen.

Bei der primären Diagnostik ist neben konventionellem Röntgen eine erweiterte Diagnostik mit Magnetresonanztomographie (MRT) und Computertomographie (CT) durchzuführen.

Aufgrund der erhöhten Strahlenbelastung sollte die Indikation zur Magnetresonanztomographie (MRT) nur bedingt gestellt werden.

Eine konventionelle Ganzbeinstandaufnahme ist wichtig zur Beurteilung des SLOPE-Winkels.

Eine 23-jährige Frau hat sich vor 3 Tagen beim Fußballspielen das linke Kniegelenk verletzt. In der Magnetresonanztomographie (MRT) wird eine Läsion des vorderen Kreuzbandes ohne Begleitverletzungen beschrieben. Welches weitere Vorgehen ist sinnvoll?

Individuelle Entscheidung zur konservativen oder operativen Therapie in Abhängigkeit der subjektiven und objektiven Instabilität des Kniegelenkes

Umgehende Vorbereitung zur operativen Versorgung binnen 24 h mittels Kreuzbandersatzplastik

Durchführung einer Computertomographie (CT) mit Rotations- und Achsanalyse als erweiterte Diagnostik

Umgehende Vorbereitung zur operativen Versorgung binnen 24 h mittels Kreuzbandnaht/-refixation, da alle frischen Kreuzbandverletzungen grundsätzlich banderhaltend operiert werden sollten

Einleiten einer konservativen Therapie, da ohne Begleitverletzungen eine operative Therapie nicht notwendig ist

Sie haben bei einem 27-jährigen Freizeithandballspieler eine vordere Kreuzbandruptur mit begleitender Korbhenkelläsion des Innenmeniskus festgestellt. Das Trauma liegt 5 Tage zurück. Das Kniegelenk ist weitgehend abgeschwollen. Das Bewegungsausmaß beträgt nach Neutral-Null-Methode Flexion/Extension: 60°-20°-0°. Die Indikation zur operativen Versorgung wurde gestellt. Welches Vorgehen erscheint Ihnen am geeignetsten?

Die Operation wird elektiv ca. 6 Wochen nach Trauma geplant, wenn der Patient ein Bewegungsausmaß nach Neutral-Null-Methode von Flexion/Extension: 130°-0°-0° erreicht hat.

Sie planen eine zweizeitige Operation mit primärer Refixation der Innenmeniskuskorbhenkelläsion und ca. 6 Wochen nach Trauma eine Stabilisierung mittels vorderer Kreuzbandersatzplastik, wenn der Patient ein Bewegungsausmaß nach Neutral-Null-Methode von Flexion/Extension: 90°-0°-0° erreicht hat.

Sie planen eine zweizeitige Operation mit medialer Meniskektomie, um möglichen Gelenkblockaden vorzubeugen, und ca. 6 Wochen nach Trauma eine Stabilisierung mittels vorderer Kreuzbandersatzplastik, wenn der Patient ein Bewegungsausmaß nach Neutral-Null-Methode von Flexion/Extension: 90°-0°-0° erreicht hat.

Es wird unter dem Hinweis auf eine mögliche erhöhte Arthrofibroserate eine zeitnahe Operation mit primärer Refixation der Innenmeniskuskorbhenkelläsion und Stabilisierung mittels vorderer Kreuzbandersatzplastik oder Naht/Refixation geplant, um die Chancen einer suffizienten Ausheilung des Innenmeniskus bei stabilisiertem Kniegelenk zu erhöhen.

Sie leiten eine konservative Therapie ein, um zu evaluieren, ob die Meniskusläsion symptomatisch und die vordere Kreuzbandruptur subjektiv instabil ist.

Eine 73-jährige Rentnerin stellt sich bei Ihnen mit medialseitigen Kniegelenkschmerzen, Schwellneigung und leichter subjektiver Instabilität vor. Sie sei vor 2 Wochen beim Spazieren mit dem Knie „weggeknickt“. Davor habe sie kaum Probleme gehabt. In der eingeleiteten Diagnostik mittels konventionellen Röntgens und Magnetresonanztomographie (MRT) zeigen sich ein flächiger medialer Knorpelschaden Grad 3 bis stellenweise 4 nach Outerbridge sowie eine Femoropatellararthrose. Das vordere Kreuzband zeigt sich mukoid degeneriert und elongiert. Die Reste des degenerierten Innenmeniskus sind bei transmuralem Vertikalriss im Hinterhorn nach medial extruiert. Außer einem halbstündigen täglichen Spaziergang besteht kaum körperliche Aktivität. Sie ist kardial stark vorerkrankt. Welche Therapie ist am ehesten zu empfehlen?

Eine valgisierende Beinachsenkorrektur, Knorpelzelltransplantation und vordere Kreuzbandersatzplastik

Eine isolierte vordere Kreuzbandersatzplastik mit Innenmeniskusteilresektion

Zeitnahe Planung eines endoprothetischen Kniegelenkersatzes

Einleitung einer konservativen Therapie

Empfehlung, zu Hause zu bleiben und auf das Spazieren zu verzichten

Sie planen bei einem sportlichen 25-jährigen Fliesenleger eine primäre vordere Kreuzbandersatzplastik. Welche Aussagen zu den Transplanten, deren Fixation und der Operationstechnik sind richtig?

Das Einlegen des Transplantates in Vancomycin-Lösung sollte nur bei autologen Transplantaten (Spendersehnen) erfolgen.

Die Quadricepssehne ist in diesem Fall nicht geeignet, da die Transplantatbreite zu gering wäre.

Die transtibiale femorale Bohrkanalanlage kann als Goldstandard bezeichnet werden.

Bei Fliesenlegern sollte immer in Doppelbündeltechnik mit gelenkferner Fixation operiert werden, da dieses Verfahren bezüglich der Ergebnisse allen anderen überlegen ist.

Auf die Verwendung der Patellasehne sollte bei Patienten mit kniender Tätigkeit verzichtet werden.

Eine 28-jährige Snowboard-Kader-Athletin hat innerhalb von 3 Jahren die zweite Rezidivruptur des linken vorderen Kreuzbandes erlitten. Auch am rechten Kniegelenk wurde schon eine Kreuzbandplastik durchgeführt; diese ist intakt. Ihnen liegen die Operationsberichte der beiden Kreuzbandoperationen am linken Kniegelenk sowie eine aktuelle Magnetresonanztomographie (MRT) vor. Auf welche weiteren diagnostischen Maßnahmen kann man am ehesten verzichten?

Durchführung einer Computertomographie (CT) zur Bestimmung der Bohrkanallage und -weite

Durchführung einer Computertomographie (CT) der Gegenseite, um die Bohrkanäle zu vergleichen

Erstellung eines konventionellen seitlichen Röntgenbildes zur Bestimmung des SLOPE-Winkels

Anfertigung einer Ganzbeinstandaufnahme zum Ausschluss einer höhergradigen Varusfehlstellung

Abklärung der noch vorhandenen Transplantate aufgrund der erfolgten Voroperationen

Was ist gegenwärtiger Wissensstand bezüglich der unterschiedlichen Operationstechniken?

Die unterschiedlichen Techniken sollten in Abhängigkeit des Alters der Patienten angewendet werden.

Die Langzeitergebnisse der aktuellen Operationstechniken zur Refixation bzw. Naht am vorderen Kreuzband sind deutlich schlechter als bei der Ersatzplastik.

Die Refixation bzw. Naht des vorderen Kreuzbandes ist ein hochkomplexer Eingriff mit deutlich erhöhten Materialkosten.

Sofern möglich, sollte der originale Kreuzbandstumpf erhalten werden, da sich hierbei Vorteile bei der Propriozeption und Vaskularisierung zeigen.

Die zusätzliche anterolaterale Stabilisierung spielt nach neueren Erkenntnissen kaum noch eine Rolle für vordere Kreuzbandinstabilität.

Eine 22-jährige Speedkletterin hat von Ihnen eine VKB(vorderes Kreuzband)-Ersatzbandplastik erhalten. Was können Sie ihr hinsichtlich Nachbehandlung und Rückkehr zur Aktivität sagen?

Die Nachbehandlung erfolgt nach einem zeitlich strikt vorgegebenen Plan.

Kriterienbasierte Testverfahren sind für die Entscheidung, ob ein „return to sport/-play/-competition“ erfolgen kann, nicht geeignet.

Das Risiko von sekundären Meniskus- und Knorpelschäden ist bei erfolgreich stabilisiertem Kniegelenk geringer als bei persistierend instabilen Gelenken.

Etwa 95 % der Patienten erreichen wieder ihr ehemaliges sportliches Niveau.

Profiathleten haben ein schlechteres Outcome in Bezug auf das Erreichen des ehemaligen sportlichen Niveaus.
